# NK-cell and T-cell functions in patients with breast cancer: effects of surgery and adjuvant chemo- and radiotherapy

**DOI:** 10.1038/sj.bjc.6603840

**Published:** 2007-06-05

**Authors:** F Mozaffari, C Lindemalm, A Choudhury, H Granstam-Björneklett, I Helander, M Lekander, E Mikaelsson, B Nilsson, M-L Ojutkangas, A Österborg, L Bergkvist, H Mellstedt

**Affiliations:** 1 Immune and Gene Therapy Laboratory, Cancer Centre Karolinska, Karolinska University Hospital, SE-17176 Stockholm, Sweden; 2 Department of Oncology, Central Hospital, SE-72189 Västerås, Sweden; 3 Department of Clinical Neuroscience, Osher Center for Integrative Medicine and Section of Psychology, Karolinska Institutet, SE-17177 Stockholm, Sweden; 4 Department of Cancer Epidemiology, Karolinska University Hospital, SE-17176 Stockholm, Sweden; 5 Centre for Clinical Research, Uppsala University, Central Hospital, SE-72189 Västerås, Sweden; 6 Departments of Hematology and Oncology, Karolinska University Hospital, SE-17176 Stockholm, Sweden; 7 Department of Surgery and Centre for Clinical Research, Uppsala University, Central Hospital, SE-72189 Västerås, Sweden

**Keywords:** breast cancer, Her-2, T-cell signalling proteins, cytokines, NK cytotoxicity

## Abstract

Breast cancer is globally the most common malignancy in women. Her2-targeted monoclonal antibodies are established treatment modalities, and vaccines are in late-stage clinical testing in patients with breast cancer and known to promote tumour-killing through mechanisms like antibody-dependent cellular cytotoxicity. It is therefore increasingly important to study immunological consequences of conventional treatment strategies. In this study, functional tests and four-colour flow cytometry were used to detect natural killer (NK)-cell functions and receptors as well as T-cell signal transduction molecules and intracellular cytokines in preoperative breast cancer patients, and patients who had received adjuvant radiotherapy or adjuvant combined chemo-radiotherapy as well as in age-matched healthy controls. The absolute number of NK cells, the density of NK receptors as well as *in vitro* quantitation of functional NK cytotoxicity were significantly higher in preoperative patients than the post-treatments group and controls. A similar pattern was seen with regard to T-cell signalling molecules, and preoperative patients produced significantly higher amounts of cytokines in NK and T cells compared to other groups. The results indicate that functions of NK and T cells are well preserved before surgery but decrease following adjuvant therapy, which may speak in favour of early rather than late use of immunotherapeutic agents such as trastuzumab that may depend on intact immune effector functions.

Breast cancer (BC) is the most common malignancy in women and the principal cause of death from cancer among women globally (Bray *et al*, 2004). Despite impressive progress in diagnosis and treatment (Hudis, 2003), a substantial fraction of women fail conventional treatments (i.e. surgery, radiation treatment and/or chemotherapy) and ultimately relapse and die (Cheng *et al*, 2004). Her-2 targeting monoclonal antibodies such as trastuzumab, given as adjuvant therapy, have significantly improved the relapse-free survival of breast cancer patients (Chan *et al*, 2006). However, it is not yet clear whether adjuvant trastuzumab treatment ought to be incorporated early, that is together with chemotherapy, or late, that is after end of adjuvant chemo-radiotherapy (Gennari *et al*, 2004). In addition to their direct apoptosis-inducing effect on tumour cells via the antagonistic effect on critical tumour cell receptors, trastuzumab is known to promote immune-mediated destruction of tumour cells by mechanisms such as antibody-dependent cellular cytotoxicity (ADCC) (Arnould *et al*, 2006), the mechanisms of which may be critically dependent on a functioning immune system. Additional immune function-dependent modalities for treatment of breast cancer that are currently in development include vaccines that induce active immunity against the cancer in patients (Yi *et al*, 2006). Thus, the above approaches to adjuvant therapy of breast cancer are impingent on the preservation of immune function in the patient during and following tumour debulking procedures.

Previous studies have reported that functional activity of natural killer (NK) cells is decreased and these cells undergo spontaneous apoptosis in patients with cancer (Konjevic and Spuzic, 1992; Bauernhofer *et al*, 2003). In contrast, increased NK-cell numbers and NK activity have also been reported in breast cancer and colon cancer before neo-adjuvant immunotherapy and correlated to time to treatment failure (Nicolini *et al*, 1996; Murta *et al*, 2000; Liljefors *et al*, 2003). Abnormalities in expression of activation molecules such as TCR , ZAP70 and p56 lck protein expression using western blot in BC (Kurt *et al*, 1998) as well as impaired expression of interferon-*γ* (IFN-*γ)* in BC and lung cancer using enzyme-linked immunosorbent assay (Caras *et al*, 2004) have been described. However, to our knowledge no studies have examined the effects of conventional adjuvant breast cancer therapy on the immune function in these patients. Given the emerging adjuvant use of trastuzumab (as well as future vaccines), the present study was conducted to investigate the NK- and T-cell function in breast cancer patients before and after primary treatment with surgery and adjuvant chemo-radiotherapy with the intention of delineating any potential immune aberrations that may result from therapy. The results would be beneficial in providing a scientific basis to integrate adjuvant immunotherapy in an optimal way in such patients.

## MATERIALS AND METHODS

### Patients and treatments

Fifty women with BC and 11 healthy age-matched controls were included. The patients were all treated in the same surgery and oncology unit, according to the National Treatment Guidelines. Patients were treated according to the Helsinki declaration on the participation of human subjects in medical research and all blood samples were collected between 0800 and 0900 hours (to minimise diurnal influence) after obtaining informed consent as per protocols approved by the local ethical committee. Patient characteristics are shown in Table 1.

Blood samples from pretreatment (*n*=9) (PT group) was drawn 1–3 days before surgery and from post-treatment patients (*n*=41) (RT, RT+CT groups). Median time was 45 (5–100) and 120 (54–164) days after finishing adjuvant therapy.

About 50 Gy total radiation, fractioned in 2 Gy doses was delivered to the breast. Patients with lymph node involvement (*n*=18) had additional radiation delivered to the adjacent lymph nodes. Twenty patients received radiotherapy alone (RT group) and 21 received a combination of radiation and chemotherapy (RT+CT group) with 5-fluorouracil, epirubicin and cyclophosphamide (FEC). Comparable number of patients in both arms received adjuvant tamoxifen therapy at the time of testing.

### Monoclonal antibodies and other reagents

Antibodies conjugated with FITC, PE, PerCP or APC against the surface molecules CD3, CD4, CD8, CD19, CD25, CD28, CD56, CD94, CD161 and NKB1 and the cytokines IFN-*γ*, IL-2 and IL-4, as well as isotype-matched negative controls were commercially purchased from Becton-Dickinson (BD) (Mountain view, CA, USA). Mabs against the signal transduction molecules P56^lck^, p59^fyn^, Zap70 and PI3k were purchased from Transduction Laboratories (Lexington, KY, USA) and antibody against CD3*ζ* from Bio Site (Stockholm, Sweden). Saponin, PMA and ionomycin were purchased from Sigma (St Louis, MO, USA) and Brefeldin A and goat antimouse Mab were obtained from BD.

### Isolation of peripheral blood mononuclear cells and cell culture conditions

Peripheral blood mononuclear cells (PBMNC) were isolated from heparinised blood by separation on a Ficoll–Isopaque gradient (Amersham Pharmacia Biotech AB, Uppsala, Sweden). To assess the ability of T and NK cells to produce cytokines in response to stimuli, 1–2 × 10^6^ cells ml^−1^ were stimulated with 25 ng ml^−1^ PMA and 1 *μ*g ml^−1^ ionomycin in RPMI-1640 medium containing 10% heat-inactivated fetal calf serum (FCS), 2 mM l^−1^ glutamine, 100 U ml^−1^ penicillin, 100 *μ*g ml^−1^ streptomycin (Gibco BRL, Paisley, UK) and 10 *μ*g ml^−1^ Brefeldin A in Falcon tubes. Unstimulated samples were set up in parallel, but without PMA and ionomycin. The Falcon tubes were incubated at 37°C in a 5% CO_2_ for 4 h.

### Cellular staining and flow cytometry

Flow-cytometric analyses were carried out using a FACSCalibur (BD) as described in a previous study (Mozaffari *et al*, 2004). Fresh cells were used whenever possible, failing which cryopreserved cells, ficolled to remove dead cells and debris, were used. Briefly, 5 × 10^5^ cells per tube were incubated with the appropriate concentration of antibodies or isotype controls (2–10 *μ*l) for 30 min on ice. For indirect staining of intracellular cytokines and signalling molecules, the cells were permeabilised with 0.1% saponin and incubated with the primary antibodies or isotype controls for 30 min at room temperature in the dark. Goat anti-mouse FITC-conjugated secondary antibody was then added to the washed cells and incubated for 10 min. Flow-cytometry gating was used to detect and separate lymphocytes and to analyse the T and NK cells. Criteria for positive staining were set at fluorescent intensities displayed by <1% of the cells stained with the isotype controls. Threshold frequencies of cytokine production by unstimulated control cells were subtracted from the percentage of cytokine producing cells quantified after stimulation.

### Calculation of absolute cell numbers

Lymphocytes expressing NK receptors and T-cell subset markers as determined by flow cytometry were expressed as a percentage of the total population. To determine the number of the populations per millilitre of blood, the percentage fraction of lymphocytes was multiplied by the number of lymphocytes per litre as determined by an automated differential blood count on the same sample.

### NK- cell-mediated cytotoxicity

Natural killer cell function was measured *in vitro* using a chromium release assay. Briefly, NK-sensitive K562 cells were labelled with 100 *μ*Ci Na_2_^51^CrO_4_ (37 MBq, 1 mCi, Amersham, UK) for 1 h and cocultured with effector cells for 4 h at various effector: target cell ratios in triplicates in 96-well plates (50 : 1, 25 : 1, 12.5 : 1, 6.25 : 1). Spontaneous ^51^Cr release was determined by incubating target cells alone, and total release by lysing labelled cells with 5% Triton X-100. After incubation the supernatant was counted in a gamma counter. NK cytotoxic activity was calculated as number of lytic units (LU) per 10^6^ effector cells. One lytic unit is the number of effector cells capable of lysing 30% of the target cells and the calculations were performed using computer software kindly provided by Dr T Whiteside (University of Pittsburgh, Pittsburgh, PA, USA).

### Proliferation assay

Mononuclear cells from patients or healthy volunteers (1 × 10^5^ cells well^−1^) were incubated in medium alone, medium containing 10 *μ*g ml^−1^ phytohaemagglutinin (PHA) (Gibco BRL) or 2.5 *μ*g ml^−1^ of mycobacterial purified protein derivative (PPD) (National Serum Institute, Copenhagen, Denmark) in a 96-well culture plate. Cultures were incubated for 3 days and 1 *μ*Ci well^−1 3^H-thymidine (Amersham Pharmacia Biotech, Uppsala, Sweden) was added to each well for the final 16–18 h. Cells were harvested and the incorporated radioactivity was measured in a *β*-counter (Micro *β* 1450, Wallace, Turku, Finland). Results were reported as stimulation index, calculated as the ratio of radioactivity of cells incubated with PHA or PPD and the radioactivity of control cultures.

### Statistical methods

The Kruskal–Wallis or Mann–Whitney *U*-test was used to calculate statistical significance in expression of cell surface molecules among the four groups of patients. Simple linear regression analysis was used to explore the possible correlation between immunological parameters, for example LU and NK receptors. Results were considered to be statistically significant for *P*<0.05. Data from the phenotypic analyses as well as functional assays from healthy donors and patients had a normal distribution pattern.

## RESULTS

### NK cells

**Figure 1:**
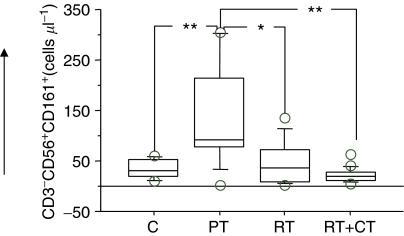
Absolute number of CD161^+^NK cells in normal control donors (C) (*n*=11), pretreatment (PT) (*n*=9), post-treatment radiation (RT) (*n*=20) and post-treatment radiation/chemotherapy (RT+CT) (*n*=21) treated breast cancer patients. The box represents the 25th to 75th percentiles. The line in the middle of the box represents the median. The top whisker is drawn from the value associated with the 75th to the 90th percentile, the bottom one associated with the 25th to the 10th percentile. The outliers show the highest and lowest 10% of observed values. ^*^P< 0.05, ^**^P< 0.01 and ^***^P< 0.001.

**Figure 2:**
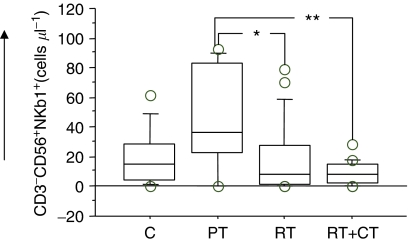
Absolute number of NKB1^+^NK cells in normal control donors (C) (*n*=11), pretreatment (PT) (*n*=9), post-treatment radiation (RT) (*n*=20) and post-treatment radiation/chemotherapy (RT+CT) (*n*=21) treated breast cancer patients. For symbols see Figure 1.

Four-colour flow cytometry demonstrated significantly higher absolute numbers of NK cells (CD3^−^CD56^+^) in the PT group compared to RT and RT+CT groups as well as to healthy controls. In particular, the RT+CT group had significantly lower number of NK cells than all other groups (Table 2). The frequency of cells expressing the NK-cell receptors CD161 (Figure 1), NKB1 (Figure 2) and CD94 (not shown) demonstrated the same pattern. There was also a significantly higher NK cytotoxicity in the PT group in comparison to post-treatment group and healthy volunteers, with the lowest cytotoxicity observed in the RT+CT group and in healthy volunteers (Table 2).

Intracellular staining for IFN-*γ* in NK cells revealed comparable patterns to the LU and overall NK frequency. Significantly higher frequency of IFN*γ*-producing NK cells was found in patients before treatment compared to all other groups (Table 2).

### Analysis of regulatory T cells and T-cell surface markers

**Figure 3:**
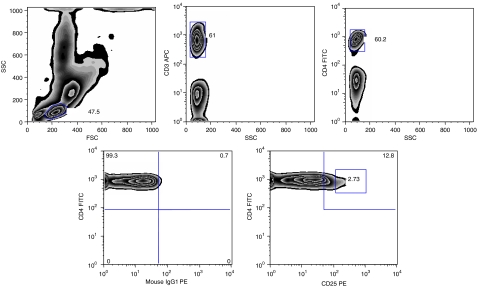
Flow cytometric analysis of CD4^+^CD25^high^ and CD4^+^CD25^+^ T cells in healthy donors and breast cancer patients. A minimum of 20 000 events in the lymphocyte gate was calculated during acquisition and the analyses was performed on all events calculated. Numbers in the plot indicate the percentage of cells within the respective rectangle.

**Figure 4:**
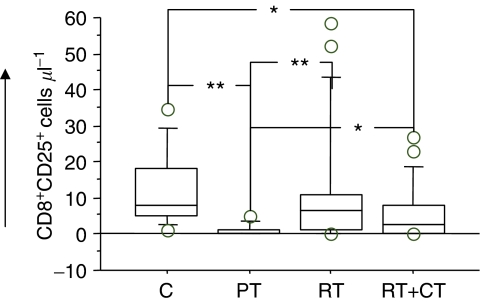
Absolute number of CD25^high^ expressing CD8 T cells in normal control donors (C) (*n*=11), pretreatment (PT) (*n*=9), post-treatment radiation (RT) (*n*=20) and post-treatment radiation/chemotherapy (RT+CT) (*n*=21) treated breast cancer patients. For symbols see Figure 1.

**Figure 5:**
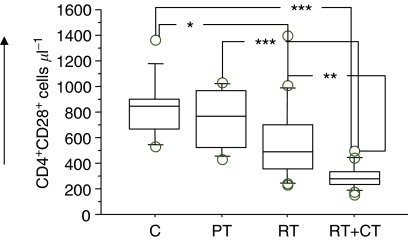
Absolute number of CD28 expressing CD4 T cells in normal control donors (C) (*n*=11), pretreatment (PT) (*n*=9), post-treatment radiation (RT) (*n*=20) and post-treatment radiation/chemotherapy (RT+CT) (*n*=21) treated breast cancer patients. For symbols see Figure 1.

Both RT and RT+CT had a profound effect in decreasing the numbers of circulating T cells with CD4^+^ cells being affected to a greater extent than CD8^+^ cells (Table 2). We subsequently analysed the Treg subsets in the patients before and after therapy in comparison to normal donors and also examined the activation markers on T cells. Tregs were measured as frequency of CD3+CD4+CD25^hi^ cells (Figure 3). The frequency of CD4+CD25^hi^ as well as CD8+CD25^hi^ T cells was significantly reduced in pretreatment patients (*P*<0.05). There was however a significant increase in CD8+CD25^hi^ in patients after adjuvant therapy compared to pretreatment (Figure 4). The fraction of CD28^+^ T cells did not differ between patients before treatment and healthy volunteers. However, patients who had received therapy as in particular the RT+CT group had a significantly reduced number of CD4+CD28+ T cells (Figure 5).

### Intracellular T-cell signalling molecules

**Figure 6:**
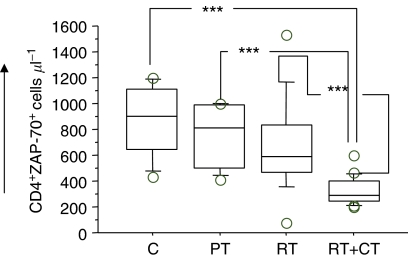
Absolute number of Zap70 expressing CD4 T cells in normal control donors (C) (*n*=11), pretreatment (PT) (*n*=9), post-treatment radiation (RT) (*n*=20) and post-treatment radiation/chemotherapy (RT+CT)(*n*=21) treated breast cancer patients. For symbols see Figure 1.

**Figure 7:**
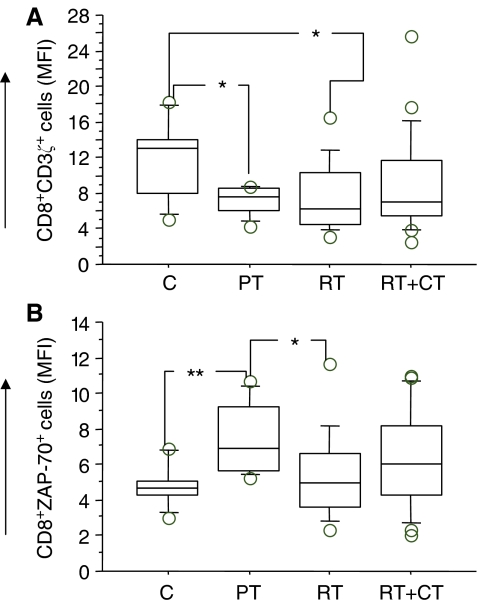
CD3*ζ* (**A**) and Zap70 (**B**) expressing (MFI) CD8 T cells in normal control donors (C) (*n*=11), pretreatment (PT) (*n*=9), post-treatment radiation (RT) (*n*=20) and post-treatment radiation/chemotherapy (RT+CT) (*n*=21) treated breast cancer patients. For symbols see Figure 1.

Patients tested before surgery had similar number of T cells expressing various signalling molecules as the control donors, whereas post-treatment patients, particularly in the RT+CT group, had a significantly reduced number of CD4^+^ cells expressing Zap70, CD3*ζ*, P56^lck^, p59^fyn^ and PI3k. There were no significant differences in CD8^+^ T cells expressing signalling molecules between the groups (data not shown). An example (ZAP70) of the staining pattern of intracellular T-cell signalling molecules in CD4T cells is shown in Figure 6. In addition to frequency of cells, the intensity of expression of the intracellular molecules was also examined using the MFI (mean fluorescence intensity) criterion. In this analysis, these were only marginal differences between the patients groups and controls as exemplified in Figure 7A (CD8+CD3*ζ*+ cells) and B (CD8+ZAP70+ cells).

### Intracellular T-cell cytokines

**Figure 8:**
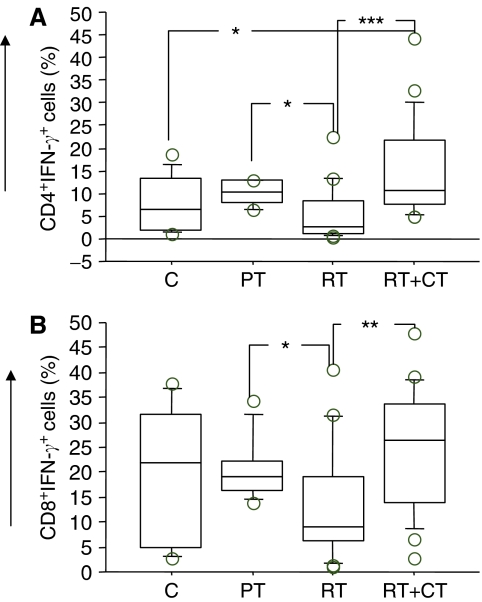
Percentage of IFN-*γ*-producing T cells in normal control donors (C) (*n*=11), Pretreatment (PT) (*n*=9), post-treatment radiation (RT) (*n*=20) and post-treatment radiation/chemotherapy (RT+CT) (*n*=21) treated breast cancer patients. (**A**) CD4; (**B**) CD8 T cells. For symbols see Figure 1.

**Figure 9:**
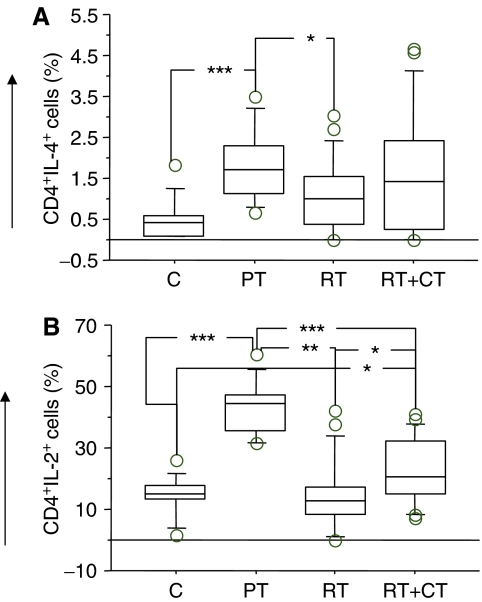
Percentage of IL-4 (**A**) and IL-2 (**B**) producing CD4 T cells in normal control donors (C) (*n*=11), pretreatment (PT) (*n*=9), post-treatment radiation (RT) (*n*=20) and post-treatment radiation/chemotherapy (RT+CT)(*n*=21) treated breast cancer patients. For symbols see Figure 1.

Frequency of IFN-*γ*, IL-4 and IL-2 producing CD4 and CD8T cells was analysed by intracellular staining and flow cytometry following activation of the T cells with PMA/ionomycin. As shown in Figure 8A (CD4) and B (CD8), there was no major difference in IFN-*γ*-producing T cells in patients compared to healthy volunteers, even though patients in RT group demonstrated some reduction in the frequency of IFN-*γ*-producing T cells. The frequency of IL-4-producing CD4 cells was significantly higher in patients before treatment compared to healthy volunteers (*P*< 0.001) and patients who received RT (*P*<0.05) (Figure 9A). There was no significant differences in IL-4-producing CD8T cells between the groups (data not shown). The pattern of IL-2 production was similar to that of IL-4 and a significantly higher level of IL-2-producing CD4T cells were detected in patients before therapy compared to all other groups (*P*<0.01 to <0.05). There were no significant differences between the groups regarding CD8T cell healthy volunteers (*P*<0.01) (Figure 9B). Similar results were observed in CD8T cells producing IL-2 (data not shown).

### PPD and PHA T-cell response

There was no difference in response to PPD between healthy volunteers and patients before therapy; however, PPD response was significantly lower in the RT group, when compared to patients before treatment (*P*<0.05). Although an increased level of PHA-induced proliferation response was seen between pretreatment patients and healthy volunteers, the observed difference was not statistically significant (data not shown).

## DISCUSSION

There is a great need to develop approaches for primary therapy of solid tumours that preserve immune function since ADCC-promoting therapeutic antibodies, such as trastuzumab and anticancer vaccination, are being increasingly developed as adjuvant treatment modalities (Arnould *et al*, 2006). With this objective in mind, this study aimed to examine the various aspects of NK- and T-cell functions in pretreatment and post-adjuvant treatment of breast cancer patients.

Untreated breast cancer patients demonstrated increased levels of NK cells in comparison to healthy subjects, but the levels were markedly reduced following therapy in both RT and RT+CT patients. The upregulation was manifested as increased frequency of CD3^−^CD56^+^ cells and increased lytic activity against K562 targets. Additionally the NK markers CD162, NKB1, CD94 and the number of IFN-*γ*-producing NK cells were increased in pretreatment patients which decreased post-therapy. Since lymphocyte counts in pretreatment patients and healthy volunteers were not significantly different, it appears that both the numbers and functional activity of NK cells were increased in patients before treatment. The augmented NK-cell cytotoxicity may be a result of activation of the innate immune system by the malignant process and/or is a result of defective regulation of NK cells in the patients. NK cells are the principal mediators of ADCC (Henney *et al*, 1981; Rosenberg *et al*, 1987). Recent work has provided further rationale for maintaining NK function in patients, especially those eligible for monoclonal antibody-based therapeutic regimens. ADCC has been demonstrated to be an important mechanism for the *in vivo* effects of antibodies like trastuzumab and rituximab (Clynes *et al*, 2000). Although NK cells are important effectors of ADCC, their ability to function in ADCC may be reduced with advanced malignancy (Kono *et al*, 2002) as well as following radio-chemotherapy (present study), possibly owing to defective expression of NK cell-triggering receptors (Costello *et al*, 2002). Our data and these previously published studies suggest the use of early, rather than late, adjuvant therapy treatment protocols for patients with breast cancer.

As expected, patients had a significant decrease in circulating T cells after therapy, with helper T cells being affected to a greater extent than cytotoxic T cells. However, the low numbers of circulating CD4^+^CD25^high^ as well as CD8^+^CD25^high^ in patients before treatment was a surprising finding and in contrast to earlier reports (Liyanage *et al*, 2002), showing an increased proportion of CD4^+^/CD25^+^ cells in breast and pancreatic cancer patients. Some of the contributing factors leading to the discrepant results may be diverse patient characteristics as well as the fact that we calculated the results as absolute number of the cells rather than percentage frequency. The increased production of IL-2 following *in vitro* stimulation of cells from patients before treatment may also be due to the low absolute numbers of CD4^+^CD25^+^ T cells and, consequently, lesser inhibitory activity. The absolute numbers of CD4^+^CD25^+^ T cells as well as CD4^+^CD25^high^ did not change significantly following treatment but a significant increase in the number of CD8^+^CD25^+^ T cells as well as CD8^+^CD25^high^ was observed compared to patients before therapy. It is interesting to notice that RT has an effect on increasing the absolute number of CD4^+^CD25^high^ and specifically on CD8^+^CD25^high^. CD8^+^CD25^high^ cells are also known to serve as regulatory T cells quite like their CD4^+^CD25^+^ counterparts (Bisikirska *et al*, 2005; Maggi *et al*, 2005).

An interesting observation was that RT+CT treatment had better preservation of IFN-*γ* secretion by CD4^+^ and CD8^+^ cells as well as better preservation of IL-2 secretion by CD4^+^ cells compared to RT arm. While the exact mechanism is not known, studies in animal models have shown that a single administration of cyclophosphamide biases the immune system in favour of a Th1 response and facilitates the secretion of cytokines such as IL-2 and IFN-*γ* (Matar *et al*, 2002). The striking similarity of our results demonstrating that RT+CT patients had higher levels of IL-2 and IFN-*γ*-secreting T cells leads us to speculate that the cyclophosphamide included in the FEC regimen may be responsible for this effect.

The ability of T cells to transduce signals to the nucleus following engagement of the TCR is a key component in the initiation of an immune response. The intracellular downstream signalling molecules, CD3*ζ*, Zap70, P56^lck^, p59^fyn^ and PI3k, were significantly reduced in the patients following treatment and the reduction was more pronounced in radiation/chemotherapy patients. There was no significant difference in frequencies of signalling molecules in patients before therapy, compared to healthy volunteers. However, the intensity of CD3*ζ* expression in CD8T cells measured as MFI was significantly downregulated. Zap70 showed a significant upregulation in T cells in patients before treatment, compared to healthy volunteer, which further contributed to the defective T-cell function in breast cancer patients. Our result support and extend the previous findings of Kurt *et al* (1998), who demonstrated decreased P56^lck^, CD3*ζ* and Zap70 protein expression in 4 of 14 breast cancer patients. However, in contrast to our study, 10 of 14 patients were in stage IV disease and had been previously treated with chemotherapy or hormonal therapy. Moreover, the signalling molecules were examined in our study using flow cytometry, a technique known to be more sensitive and quantitative compared to western blotting used in the report of Kurt *et al* (1998). Our data also indicate that T-cell function was negatively affected by treatment, especially in the RT+CT group. It has been previously shown that cytotoxic T-cell number correlate with clinical prognosis in breast cancer patients (Blake-Mortimer *et al*, 2004) and that localised radiation can cause transiently a systemic effect on T-cell function (Berger *et al*, 1990). Treatment strategies dependent on the integrity of T-cell function, such as vaccination approaches (Morse, 2000), could potentially be more efficacious if initiated before radio-chemotherapy, or following recovery of T-cell function after cessation of therapy.

Current research on immunotherapy for cancer mainly focuses on optimal use of monoclonal antibodies and on generation of T-cell-mediated immunity by vaccination strategies. Thus, the negative effects of radiation and/or chemotherapy on NK- and T-cell activity in breast cancer are worth considering when new antibody and vaccine trials are designed in patients with breast cancer.

## Figures and Tables

**Table 1:** Patient characteristics

		**Post treatment^b^ (*n*=41)**	
**Group**	**Pretreatment^a^ (*n*=9)**	**RT (*n*=20)**	**RT+CT (*n*=21)**
Age (range)	(43–75)	(52–80)	(38–69)
*Type of surgery*			
Mastectomy	—	3	2
Breast conservation	—	17	19
*Lymph nodes*			
Axillary clearance	0	5	14
Sentinel node	8	11	7
No axillary dissection	1	4	0
Cancer *in situ*	1	2	0
*No.* *of involved nodes*			
⩽3	0	0	11
4–8	1	1	4
⩾9	0	0	1
negative	8	15	5
ER+	8	17	14
PR+	8	10	9
HER2+		1	4
HER2−		1	6
Tumour size mm, median (range)		14 (5–50)	17 (7–30)
<2 cm	7	13	16
⩾2 cm	2	7	5
Adjuvant tamoxifen	5	11	15

ER+=estrogen receptor positive; HER2+=human epidermal growth factor receptor 2; PR+=progestron receptor positive.

a Blood samples were collected 1–3 days before primary surgery.

b Blood samples collected after a median time 40 days.

**Table 2:** Lymphocyte subsets in patients and healthy donors

	**C**	**PT**	**RT**	**RT+CT**
WBC^a^	6.9 × 10^3^±0.4 (5.6–10 × 10^3^)	7 × 10^3^±0.5 (4.5–9.2 × 10^3^)	5.7 × 10^3^±0.3 (4.1–8.5 × 10^3^)	4.3 × 10^3^±0.2 (3.2–5.3 × 10^3^)
Lymphocyte^b^	2 × 10^3^±0.2 (1.1–3 × 10^3^)	1.9 × 10^3^±0.1 (1.3–2.2 × 10^3^)	1.7 × 10^3^±0.1 (0.8–3 × 10^3^)	1.1 × 10^3^±0.1 (0.6–1.7 × 10^3^)
CD3^+^CD4^+^ c	858±107 (385–1512)	782±77 (435–1045)	625±79 (230–1572)	336±24 (198–606)
CD3^+^CD8^+^ d	371±76 (133–885)	260±38 (102–399)	244±32 (95–447)	311±39 (87–476)
CD3^−^CD56^+^ e	160±32 (49–410)	293±47 (74–462)	144±23 (10–428)	84±12 (7.7–150)
IFN-*γ*+NK cell frequency^f^	2.1±0.6 (0.0–6.5)	6.2±0.8 (2–9.8)	3.5±0.6 (0.1–10)	4.4±0.6 (1–11)
LU^g^	2.6±0.4 (1–6.6)	13±2.9 (1.9–32.5)	6.6±1.2 (1.3–19.5)	4.4±0.6 (0.3–10.4)

Absolute number (mean±s.e.m.) of WBC, lymphocytes, CD4/CD8T cells, NK cells per microlitre. Frequency of NK cells expressing IFN-*γ* and NK lytic unit in healthy donors (C) and patient groups before treatment (PT) after radiation therapy (RT) and after radiation+chemotherapy (RT+CT).

a The difference between the healthy donors and post-treatment group RT+CT were significant at *P*⩽ 0.001. The difference between pretreatment and post-RT was significant at *P*⩽0.05. The difference between PT and RT+CT as well as between RT and RT+CT was significant at *P*⩽0.001.

b The difference between the healthy donors and post-treatment group RT+CT were significant at *P*⩽0.01. The difference between PT and RT+CT as well as between RT and RT+CT was significant at *P*⩽0.01.

c Healthy donors, PT and RT groups did not differ significantly. All of these groups had significant difference compared to RT+CT (*P*⩽0.01–0.001).

d No significant differences between the patient groups or normal donors.

e Elevation in NK cells in PT patients compared to healthy volunteers was significant at *P*⩽0.05. RT+CT patients had significant decrease compared to healthy donors (*P*⩽0.05) as well as PT patients (*P*⩽0.001).

f Normal donors had decreased numbers of IFN-*γ-*positive cells compared to PT (*P*⩽0.01) as well as RT+CT groups (*P*⩽0.05). Decrease in RT group compared to PT was also significant (*P*⩽0.05).

g Normal donors had decreased number of lytic units compared to PT and RT groups (*P*⩽0.01–0.001). Number of lytic units in PT group was elevated compared to RT (*P*⩽0.05) as well as RT+CT groups (*P*⩽0.01).
